# Cord Blood Appetite Hormones and Early-Life Growth and Childhood Adiposity in the ENVIRONAGE Cohort

**DOI:** 10.1001/jamanetworkopen.2025.42140

**Published:** 2025-11-06

**Authors:** Thaïs De Ruyter, Nathalie Michels, Rossella Alfano, Paolo Vineis, Gary Frost, Stefaan De Henauw, Michelle Plusquin, Dries S. Martens, Tim S. Nawrot

**Affiliations:** 1 Department of Public Health and Primary Care, Ghent University, Ghent, Belgium; 2 Centre for Environmental Sciences, Hasselt University, Hasselt, Belgium; 3 Department of Developmental, Personality, and Social Psychology, Ghent University, Ghent, Belgium; 4 Department of Epidemiology and Biostatistics, School of Public Health, Imperial College London, London, United Kingdom; 5 Medical Research Council–Health Protection Agency Centre for Environment and Health, Imperial College London, London, United Kingdom; 6 Section for Nutrition Research, Department of Metabolism, Digestion, and Reproduction, Imperial College London, London, United Kingdom; 7 Department of Public Health and Primary Care, University of Leuven, Leuven, Belgium

## Abstract

**Importance:**

Understanding early-life origins of increased body mass is crucial because childhood obesity increases the risk of later development of obesity and related metabolic disorders. Appetite hormones have been investigated in relation to fat accumulation in adults, but little is known about the association between cord blood levels of these hormones and obesity-related outcomes in early childhood.

**Objective:**

To examine whether cord blood appetite hormone levels are associated with early-life growth trajectories and childhood adiposity.

**Design, Setting, and Participants:**

This prospective birth cohort study was conducted within the Environmental Influence on Early Aging (ENVIRONAGE) birth cohort, with follow-up data collected at 4 to 6 years of age. The study includes children from a population-based cohort in Belgium recruited between January 1, 2010, and December 31, 2016, with available cord blood appetite hormone levels and participating in follow-up visits.

**Exposures:**

Cord blood levels of glucagon-like peptide 1 (GLP-1), peptide tyrosine tyrosine (PYY), pancreatic polypeptide (PP), and leptin.

**Main Outcomes and Measures:**

Adiposity peak, anthropometric measures at 4 to 6 years of age (waist to height ratio and body mass index *z* score), and rapid growth and slow growth from birth to 6, 12, and 24 months.

**Results:**

A total of 325 children (median [IQR] age, 4.49 [4.04-4.95] years; 151 [46.5%] male) were studied. Analyses were performed in 2 subgroups based on availability of appetite hormones: 226 children with cord blood PP and leptin (median [IQR] age at follow-up, 4.5 [4.3-4.7] years; 108 [47.8%] male) and 229 children with cord blood PYY and GLP-1 (median [IQR] age at follow-up, 4.5 [4.3-4.8] years; 98 [42.8%] male). PYY levels were positively associated with the adiposity peak (β = 0.14; 95% CI, 0.01-0.27). PYY and PP levels were associated with an higher risk of rapid growth in the first 2 years of life (odds ratio [OR], 1.71; 95% CI, 1.03-2.84; OR, 1.65; 95% CI, 1.21-2.27, respectively). Leptin levels showed an inverse association with rapid growth (OR, 0.43; 95% CI, 0.27-0.66). PYY and PP levels were associated with a higher body mass index *z* score at 4 to 6 years of age (β = 0.16; 95% CI, 0.04-0.30; β = 0.17; 95% CI, 0.004-0.34, respectively).

**Conclusions and Relevance:**

In this cohort study of 325 children, cord blood appetite hormone levels were associated with early-life growth and adiposity, suggesting that appetite hormones may influence childhood growth and body composition trajectory from birth onward.

## Key Points

**Question:**

Are cord blood levels of appetite hormones associated with early-life growth patterns and childhood adiposity?

**Findings:**

In this birth cohort study of 325 children, cord blood peptide YY and pancreatic polypeptide levels were associated with rapid growth in early life and a higher body mass index *z* score at 4 to 6 years of age, whereas leptin levels were inversely associated with rapid growth.

**Meaning:**

These results suggest that cord blood appetite hormone levels may contribute to early childhood growth and adiposity, potentially influencing the development of obesity.

## Teaser

This cohort study of children in the ENVIRONAGE birth cohort explores the associations of a set of anorexigenic cord blood appetite hormone levels with postnatal growth, body mass index growth trajectories, and childhood adiposity measures.

## Introduction

Obesity represents a substantial global health burden, with its prevalence increasing from early childhood onward, contributing to an increased risk of noncommunicable diseases.^1^ Appetite-regulating hormones have emerged as potential molecular mechanisms underlying the development of obesity, given their critical roles in energy homeostasis and appetite control.^2^ Most appetite-regulating hormones function as satiety hormones, primarily released in the gastrointestinal tract in response to food intake to promote satiation. Their circulating levels fluctuate throughout the day in response to the body’s nutritional state, aligning energy intake with energy needs.^3^ In individuals with obesity, however, these dynamic patterns are frequently dysregulated, potentially contributing to a disrupted energy balance.^2,4,5,6^ In contrast to the episodic signals of gastrointestinal appetite hormones, leptin functions as a long-term, tonic signal of energy balance. Secreted by adipocytes, leptin reflects the body’s fat stores and provides a continuous indication of energy reserves, playing a critical role in regulating appetite and energy homeostasis.^7^

Appetite-regulating hormone levels are already detectable in cord blood of the neonate.^8,9,10,11^ Despite this, limited research has explored the functions and health implications of these hormones during early life. Notably, the circulating levels of most appetite hormones at birth and during the first postnatal weeks are much higher than those observed in adults.^8,9,10^ This finding might suggest a potential role in developmental processes, which may potentially have long-lasting effects.

Cord blood leptin levels have been associated with postnatal growth patterns and childhood body composition, with birth weight potentially acting as a mediator in this association.^12,13,14,15,16,17^ However, the findings are inconclusive.^12,13^ Similar associations for other appetite hormones remain unexplored.

Nevertheless, evidence suggests that the trajectory toward obesity is set in early life.^13,18^ The prenatal environment in particular has emerged as a key determinant of diseases in later life, potentially shaping the developmental trajectories of body composition and metabolic regulation.^19,20^ Research indicates that early growth patterns, specifically rapid infant growth and the magnitude of the adiposity peak, may be predictive of later obesity and noncommunicable diseases risk.^21,22^ These outcomes reflect critical developmental windows in body composition and metabolic regulation and are more proximal indicators of obesity in later life.

Therefore, the aim of this study was to explore the associations of a set of anorexigenic cord blood appetite hormone levels (glucagon-like peptide 1 [GLP-1], peptide tyrosine tyrosine [PYY], pancreatic polypeptide [PP], and leptin) with postnatal growth, body mass index (BMI; calculated as weight in kilograms divided by height in meters squared) growth trajectories, and childhood adiposity measures in the Environmental Influence on Early Aging (ENVIRONAGE) birth cohort. We hypothesize that higher cord blood concentrations of anorexigenic appetite-regulating hormones (GLP-1, PYY, PP, and leptin) are prospectively associated with slower postnatal growth, more favorable BMI trajectories, and lower adiposity in early childhood.

## Methods

### Study Design and Population

Participants were recruited from the ongoing ENVIRONAGE birth cohort,^23^ initiated in 2010 at East-Limburg Hospital in Genk, Belgium. The study enrolls mother-newborn pairs at delivery, excluding those with planned cesarean sections or an inability to complete questionnaires in Dutch. After 4 to 6 years, follow-up visits are conducted at the university’s examination center. The study complies with the Declaration of Helsinki^24^ and was approved by the Ethical Committees of Hasselt University and East-Limburg Hospital. Written informed consent was obtained. This study followed the Strengthening the Reporting of Observational Studies in Epidemiology (STROBE) reporting guideline for cohort studies .

**Figure 1.**
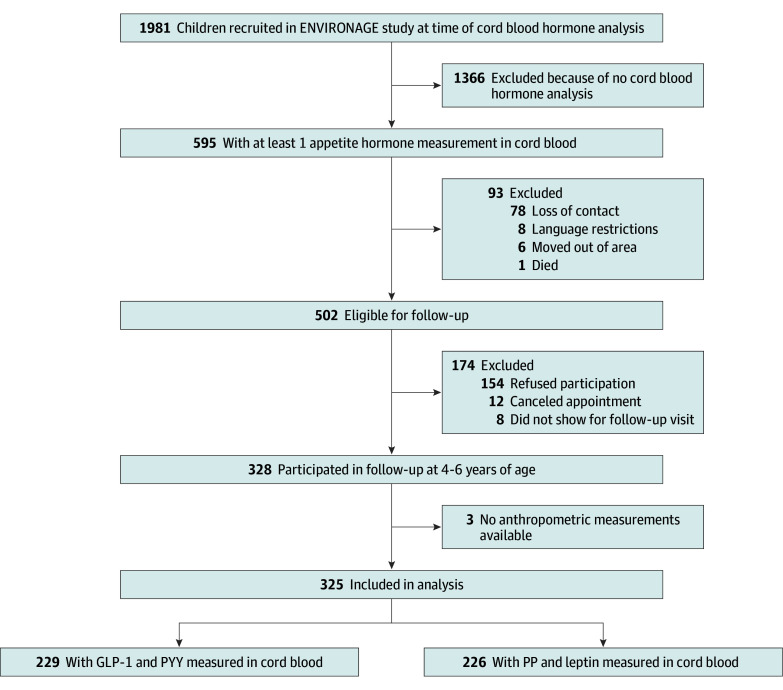
Flowchart of the ENVIRONAGE Study Participants GLP-1 indicates glucagon-like peptide 1; PP, pancreatic polypeptide; PYY, peptide tyrosine tyrosine.

This study included 325 participants (recruited from 2010 to 2016) for whom cord blood appetite hormone levels were available and participated in the follow-up visit. Based on the availability of hormone measurements, the study population can be divided into 2 subgroups: 229 children having GLP-1 and PYY measurements and 226 children having PP and leptin measurements in cord blood. A total of 130 children had all 4 hormones measured. A detailed flow diagram is presented in Figure 1. The analyzed ENVIRONAGE population is demographically similar to the nonanalyzed participants of the cohort, with slight differences in maternal education and reported alcohol use during pregnancy (eTable 1 in Supplement 1).

### Clinical Measures, Anthropometrics, and Sample Collection

After delivery, the mothers completed questionnaires, providing detailed lifestyle and sociodemographic information about the mother (eg, maternal age, race, educational level, and smoking and alcohol use during pregnancy). Race categories included Eastern European, Morocco, Southern European, Turkish, Western European, and other (any other race). Further information on the delivery and health status of the mother and newborn was derived from medical records of the hospital (eg, newborn sex, mode of delivery, and gestational age) More information is available in eMethods 1 in Supplement 1.

#### Appetite Hormone Analysis

Appetite hormone levels were measured in cord blood plasma. PYY and GLP-1 levels were measured using previously described radioimmunoassays.^25,26^ Leptin and PP levels were measured using a magnetic bead panel (EMD Millipore MILLIPLEX Human Metabolic Hormone Magnetic Bead Panel HMH3-34K, Millipore Merck). More information is provided in eMethods 2 in Supplement 1.

#### Postnatal Growth and Adiposity Measurements

Given the limited prior research on cord blood appetite-regulating hormones other than leptin, no single outcome was designated as primary. Instead, an exploratory approach was adopted examining several early-life growth and adiposity measures that are established risk factors for later obesity risk. Results should therefore be interpreted as hypothesis-generating. To monitor the children’s postnatal growth between birth and 29 months of age, mothers provided copies of the medical records from Kind en Gezin (Child and Family), the Flemish agency responsible for the health, childcare, and well-being of children up to school age. Using these data, growth patterns were characterized with 3 indicators: rapid growth, adiposity peak, and BMI growth trajectories.

Rapid growth was defined as a World Health Organization *z* score increase of more than 0.67 in weight for age between 2 time points, following the work of Ong et al.^27^ Conversely, slow growth was defined as a World Health Organization *z* score decrease of more than 0.67.^27^ Growth rates were calculated for 3 periods, birth to 6 months, birth to 12 months, and birth to 24 months, to assess time-based differences in associations. A 2-step prediction approach was used to estimate sex- and age-specific weight at 6, 12, and 24 months of follow-up, using fractional polynomials of age by gender in the ENVIRONAGE cohort, as previously described.^28^

The adiposity peak is the peak observed in a child’s BMI growth curve. Based on the predicted BMI trajectories in our cohort, using the aforementioned 2-step prediction model, the adiposity peak in the ENVIRONAGE cohort was estimated to occur at 13 months (eFigure 1 in the Supplement). Therefore, the predicted BMI at 13 months was used as a proxy for the adiposity peak, which serves as an early-life predictor for obesity later in life.^28,29,30^ BMI growth trajectories from birth up to 29 months were modeled using natural cubic splines (eMethods 3 in Supplement 1). Childhood adiposity was measured at the follow-up visit around the age of 4 years using anthropometric measures: BMI (*z* score) and waist to height ratio (eMethods 4 in Supplement 1).

### Statistical Analysis

Statistical analyses were conducted with R software, version 4.3.1 (R Project for Statistical Computing). Appetite hormone values were natural log–transformed to improve normality. Correlations between the appetite hormones were evaluated using Spearman rank correlations. Continuous data were compared with the Wilcoxon test and proportions with the χ^2^ test. A 2-sided *P* < .05 was considered statistically significant. Unless stated otherwise, all statistical analyses were performed using 2 models adjusted for a priori chosen variables. The basic model was adjusted for sex.^31,32^ The fully adjusted model further included potential confounders, including maternal age at delivery,^16^ maternal education level as a proxy for socioeconomic status,^33^ prepregnancy BMI,^34^ gestational weight gain,^35^ maternal smoking and alcohol use during pregnancy,^36,37^ and parity^38^; and additional covariates to increase model precision: mode of delivery,^39^ gestational age at delivery,^40,41^ and breastfeeding.^42,43^ Missing variables (breastfeeding, n = 34; alcohol consumption during pregnancy, n = 4; and maternal weight gain during pregnancy, n = 5) were imputed via multiple imputation, using the mice package with 13 imputations.

The associations of cord blood appetite hormone levels with adiposity peak and anthropometric measurements at the follow-up visit (waist to height ratio and BMI *z* score) were assessed using robust linear regression models (robust R package) to account for influential points identified using Cook’s distance.^44^ The models for BMI *z* score and waist to height ratio at the follow-up visit were additionally adjusted for age at follow-up visit (in months) and the quadratic term of age to allow for nonlinearity. Associations between cord blood appetite hormone levels and postnatal growth were assessed based on a categorical growth outcome (rapid, normal, and slow growth), using multinomial logistic regression models (with normal growth as the reference category).

Several sensitivity analyses were performed. First, birth weight was not included in the primary models because it could act as potential mediator, but age- and sex-standardized birth weight *z* scores were included in sensitivity analyses for all outcomes because they could also theoretically act as a confounder.^45^ Second, to examine the potential associations of gestational diabetes^46^ and preeclampsia^47,48,49^ with the results, we excluded samples of neonates born to mothers with gestational diabetes or preeclampsia. Third, interaction terms between hormone levels and sex were included in the fully adjusted models to investigate whether associations differed by sex. Associations between cord blood appetite hormone levels and BMI growth trajectories were analyzed using linear mixed-effects models (eMethods 3 in Supplement 1).

## Results

### Descriptive Statistics

A total of 325 children (median [IQR] age, 4.49 [4.04-4.95] years; 151 [46.5%] male and 174 [53.5%] female; 3 [0.9%] Eastern European, 3 [0.9%] Morocco, 9 [2.8%] Southern European, 13 [4.0%] Turkish, 294 [90.7%] Western European, 2 [0.6%] other) were studied. The characteristics of the participants included in this study stratified per group of available appetite hormones (n = 226 for leptin and PP and n = 229 for GLP-1 and PYY)^24^ are given in Table 1. There were no significant differences in characteristics between both subpopulations. Spearman rank correlations among the concentrations of the 4 cord blood hormones are shown in eFigure 2 in Supplement 1. Moderate correlations were found between PYY and GLP-1 (*r* = 0.42; *P* < .001), and PYY and PP (*r* = 0.24; *P* = .007). Unadjusted regression results of associations among study outcomes, included confounders, and cord blood appetite hormones are shown in eTable 2 in Supplement 1.

**Table 1.** Population Characteristics

Characteristic	Participants, No. (%)		*P* value^a^
	PP and leptin (n = 226)	PYY and GLP-1 (n = 229)	
Newborn			
Sex			
Male	108 (47.8)	98 (42.8)	.33
Female	118 (52.2)	131 (57.2)	
Birth weight, median (IQR), g	3360 (3030 to 3718)	3395 (3090 to 3660)	.65
Gestation duration, median (IQR), wk	39 (38 to 40)	39 (38 to 40)	.90
Preterm	15 (6.6)	13 (5.7)	.81
Maternal			
Age at delivery, median (IQR), y	30 (27 to 33)	30.0 (27.0 to 32.0)	.44
Gestational weight gain, median (IQR), kg	13.0 (10.0 to 16.7)	14.0 (11.0 to 17.5)	.30
Prepregnancy BMI	23.8 (21.4 to 26.4)	23.9 (21.5 to 26.6)	
Prepregnancy weight			
Underweight	6 (2.7)	7 (3.1)	.77
Normal weight	142 (62.8)	141 (61.6)	
Overweight	52 (23)	54 (23.6)	
Obesity	26 (11.5)	27 (11.8)	
Parity			
1	125 (55.3)	123 (53.7)	.82
2	80 (35.4)	87 (38.0)	
≥3	21 (9.3)	19 (8.3)	
Education level^b^			
Low	20 (8.8)	14 (6.1)	.46
Middle	59 (26.1)	67 (29.3)	
High	147 (65.0)	148 (64.6)	
Gestational diabetes	11 (4.9)	8 (3.5)	.62
Preeclampsia	2 (0.9)	2 (0.9)	>.99
Alcohol use during pregnancy	48 (21.6)	42 (18.3)	.45
Smoking during pregnancy	26 (11.5)	27 (11.8)	>.99
Breastfeeding	150 (73.9)	152 (74.9)	.91
Cord blood appetite hormone levels			
PYY, median (IQR), pmol/L	118.56 (80.12 to 154.14)	115.03 (81.28 to 152.71)	.91
GLP-1, median (IQR), pmol/L	34.62 (28.50 to 42.01)	35.55 (27.99 to 42.68)	.88
Leptin, median (IQR), pg/mL	13 060.75 (7222.81 to 24 145.74)	11 863.73 (5571.09 to 23 569.16)	.31
PP, median (IQR), pg/mL	24.09 (16.47 to 44.86)	23.67 (17.01 to 36.41)	.84
Child at follow-up visit			
Age, median (IQR), y	4.50 (4.28 to 4.74)	4.50 (4.29 to 4.78)	.92
Estimated BMI at 9 months, median (IQR)	17.21 (16.46 to 18.14)	17.16 (16.44 to 18.06)	.82
Growth the first 6 months			
Slow growth	73 (32.3)	70 (30.6)	.88
Normal growth	122 (54.0)	129 (56.3)	
Rapid growth	31 (13.7)	30 (13.1)	
Growth the first year			
Slow growth	46 (20.4)	41 (17.9)	.50
Normal growth	108 (47.8)	122 (53.3)	
Rapid growth	72 (31.9)	66 (28.8)	
Growth the first 2 years			
Slow growth	37 (16.4)	31 (13.5)	.65
Normal growth	86 (38.1)	94 (41.0)	
Rapid growth	103 (45.6)	104 (45.4)	
BMI *z* score, median (IQR)^c^	0.43 (−0.08 to 1.03)	0.52 (−0.09 to 1.05)	.65
Waist to height ratio, median (IQR)	0.49 (0.47 to 0.51)	0.49 (0.48 to 0.51)	.40
Overweight^d^	33 (14.6)	34 (14.8)	>.99
Obesity^d^	6 (2.7)	4 (1.7)	.73

Abbreviations: BMI, body mass index (calculated as weight in kilograms divided by height in meters squared); GLP-1, glucagon-like peptide 1; PP, pancreatic polypeptide; PYY, peptide tyrosine tyrosine.

^a^ Pearson χ^2^ test or Wilcoxon rank-sum test.

^b^ Maternal educational level was categorized by *International Standard Classification of Education* and coded as low (no diploma, primary school, or lower secondary school(middle school), middle (high school diploma), or high (college or university degree).

^c^ BMI *z* scores were calculated according to the World Health Organization’s Child Growth Standards based on length and height, weight, and age of the child.

^d^ Overweight and obesity were determined based on the cut-offs proposed by Cole et al. using BMI *z* scores.^24^

### Adiposity at 4 to 6 Years of Age

Table 2 shows the associations between the cord blood appetite hormone levels and adiposity measurements obtained at the follow-up visit. In the basic models, PP levels were positively associated with waist-to-height ratio (β = 0.16; 95% CI, 0.03-0.28; *P* = .008). In the fully adjusted models, a positive association was observed for PYY and BMI *z* score (β = 0.16; 95% CI, 0.04-0.27; *P* = .01). Excluding children born to mothers with gestational diabetes or preeclampsia revealed a positive association for PP and BMI *z* score in the fully adjusted models, which was also observed when correcting for birth weight (eTables 3 and 4 in Supplement 1). Interaction terms between sex and hormone levels were not significant.

**Table 2.** Associations Between Cord Blood Appetite Hormone Levels and Adiposity Measures at 4 to 6 Years of Age in 229 Children With GLP-1 and PYY Measurements

Hormone	Basic model^a^		Fully adjusted model^b^	
	β (95% CI)^c^	*P* value	β (95% CI)^c^	*P* value
**Waist to height ratio**				
GLP-1	−0.08 (−0.22 to 0.05)	.25	−0.08 (−0.22 to 0.06)	.24
PYY	0.05 (−0.07 to 0.18)	.40	0.06 (−0.08 to 0.21)	.36
PP	0.16 (0.04 to 0.28)	.008	0.13 (−0.007 to 0.27)	.06
Leptin	−0.0241 (−0.14 to 0.12)	.72	0.009 (−0.14 to 0.16)	.91
**BMI *z* score**				
GLP-1	−0.008 (−0.14 to 0.12)	.90	−0.005 (−0.18 to 0.17)	.95
PYY	0.11 (−0.03 to 0.25)	.13	0.16 (0.04 to 0.30)	.01
PP	0.12 (−0.04 to 0.28)	.13	0.17 (−0.004 to 0.34)	.06
Leptin	−0.05 (−0.19 to 0.08)	.43	−0.004 (−0.18 to 0.18)	.96

Abbreviations: BMI, body mass index (calculated as weight in kilograms divided by height in meters squared); GLP-1, glucagon-like peptide 1; PP, pancreatic polypeptide; PYY, peptide tyrosine tyrosine.

^a^ Basic models were adjusted for sex and age at follow-up visit.

^b^ Fully adjusted models were further adjusted for breastfeeding, gestational age, mode of delivery, maternal smoking and alcohol use during pregnancy, maternal educational level, maternal age at delivery, gestational weight gain, prepregnancy BMI, and parity.

^c^ Estimates are presented as standardized regression coefficients.

### Adiposity Peak

Cord blood PYY levels were positively associated with the adiposity peak, although only in the fully adjusted model (β = 0.14; 95% CI, 0.01-0.27; *P* = .03) (Table 3). This association remained similar when adjusting for birth weight or exclusion of children born to mothers with preeclampsia or gestational diabetes (eTables 5 and 6 in Supplement 1). Interaction terms between sex and hormone levels were not significant in any of the models.

**Table 3.** Associations Between Cord Blood Appetite Hormone Levels and the Estimated BMI at 13 Months of Age in 229 Children With GLP-1 and PYY Measurements

Hormone	Basic model^a^		Fully adjusted model^b^	
	β (95% CI)^c^	*P* value	β (95% CI)^c^	*P* value
GLP-1	0.02 (−0.15 to 0.11)	.79	0.01 (−0.12 to 0.14)	.87
PYY	0.06 (−0.07 to 0.19)	.40	0.14 (0.01 to 0.27)	.03
PP	0.07 (−0.08 to 0.22)	.38	0.10 (−0.05 to 0.25)	.18
Leptin	0.03 (−0.10 to 0.16)	.67	−0.06 (−0.08 to 0.20)	.39

Abbreviations: BMI, body mass index (calculated as weight in kilograms divided by height in meters squared); GLP-1, glucagon-like peptide 1; PP, pancreatic polypeptide; PYY, peptide tyrosine tyrosine.

^a^ Basic models were adjusted for sex.

^b^ Fully adjusted models were further adjusted for breastfeeding, gestational age, mode of delivery, maternal smoking and alcohol use during pregnancy, maternal educational level, maternal age at delivery, gestational weight gain, prepregnancy BMI, and parity.

^c^ Estimates are presented as standardized regression coefficients.

### Postnatal Growth

**Figure 2.**
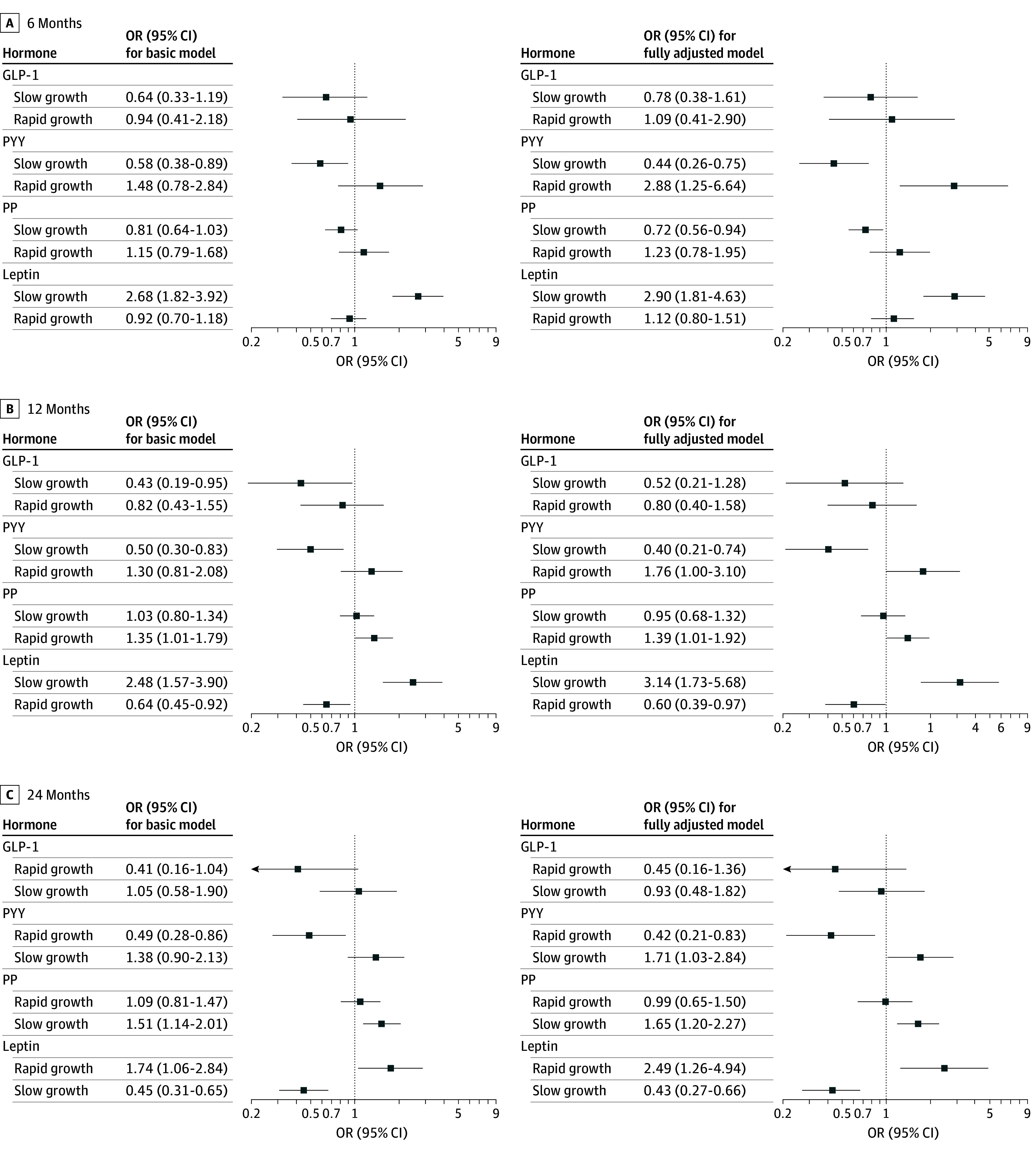
Associations Between Cord Blood Appetite Hormones and Rapid Growth Between Birth and 6 Months, 12 Months, and 24 Months Using Multinomial Regression Models Relative odds ratios (ORs; normal growth as the reference category) are represented for a doubling in cord blood hormone level. Basic models were adjusted for sex. Fully adjusted models were further adjusted for maternal age at delivery, maternal educational level, prepregnancy body mass index, gestational weight gain, maternal smoking during pregnancy, alcohol use during pregnancy, parity, gestational age at delivery, delivery method, and breastfeeding. GLP-1indicates glucagon-like-peptide-1; PP, pancreatic polypeptide; PYY, peptide tyrosine tyrosine.

Figure 2 and eTable 7 in Supplement 1 detail the associations between the cord blood hormones and postnatal growth. In general, PYY and PP levels were positively associated with rapid growth and negatively with slow growth, whereas leptin levels showed opposite associations. The basic and fully adjusted models provided similar results, except for the association between rapid growth and PYY for which the basic models were not significant. When considering the shortest time frame (birth to 6 months), only slow growth seems to be significantly associated with cord blood hormone levels, whereas in longer time frames rapid growth is associated as well, although in the opposite direction from the cord blood hormone levels. Comparable results were obtained after excluding participants with gestational diabetes or preeclampsia (eTable 8 in Supplement 1). When models were additionally adjusted for birth weight, GLP-1 was also positively associated with slow growth and negatively with rapid growth in all 3 time frames (eTable 9 in Supplement 1). Most of the associations with rapid growth were moderated by sex; sex-specific odds ratios are shown in eTable 10 in Supplement 1. Although the associations pointed in the same direction for both sexes, variations in effect size were observed.

In the spline-based mixed models, cord blood leptin was positively associated with mean BMI across childhood (β = 0.65; *P* < .001) and showed significant age interactions, indicating differences in BMI trajectory shape (eTable 11 in Supplement 1). Although PYY and PP showed no significant main associations, PYY and PP displayed age-dependent interactions (eTable 11 in Supplement 1). Predicted BMI trajectories in function of hormone levels are displayed in eFigure 3 in Supplement 1.

## Discussion

This study examined the associations of cord blood appetite hormones levels with childhood adiposity and early-life growth, both of which are known to be associated with obesity in later life. To our knowledge, this is the first study to report positive associations of cord blood PYY and PP with adiposity and rapid growth: PYY was positively associated with rapid growth, BMI *z* scores at 4 to 6 years of age, and the adiposity peak, whereas PP was associated with rapid growth and both BMI *z* scores and waist-to-height ratio at 4 to 6 years of age. Consistent with previous literature,^12,13,15,16^ we observed associations of cord blood leptin with childhood BMI trajectory and rapid growth.

### Biological and Public Health Relevance

Our findings can be contextualized within the Developmental Origins of Health and Disease hypothesis, which posits that the environment during early life, and in particular during fetal development, can have lasting impacts on health outcomes in later life.^20^ The associations observed between cord blood appetite hormones and early growth and adiposity measures suggest that hormonal environments in utero may predispose individuals to certain growth patterns, ultimately influencing their risk for obesity and related conditions in adulthood.

The findings of this study might have substantial public health implications, particularly in the context of childhood obesity and its long-term health effects. The observed results suggest that cord blood appetite hormones (PP, PYY, and leptin) might play a role in the early-life development of obesity. The positive associations of cord blood PYY and PP with BMI *z* scores at 4 to 6 years of age, when a doubling in PYY and PP levels corresponds to a 0.21 and 0.13 SD increase, respectively, are clinically relevant because previous research^50^ shows that even a 0.20 reduction in BMI *z* scores can lower cardiovascular risk factors in children. Moreover, a doubling of cord blood leptin levels was associated with a reduced likelihood of rapid growth in the first year of life, comparable to the beneficial effect of mixed breastfeeding vs formula feeding on rapid growth in the same period.^51^ These associations, although modest, indicate that even small changes in hormone levels during critical developmental windows could have substantial long-term health consequences.

### Leptin

Although negative associations between cord blood leptin levels and childhood body composition have been reported,^12,13,16,17,52^ this was not confirmed in this study. This discrepancy might arise from differences in sample size, methods, and age of the population. Previous studies suggest age-related shifts in the association between cord blood leptin and adiposity: strongly positive at birth,^53^ reversing to weaker negative associations by 3 years of age,^17,52^ and diminishing thereafter,^12,13,17^ which is in line with the current findings (4 to 6 years of age). Nevertheless, in this study, we observed significant associations with rapid growth, consistent with previous literature.^15,54^ Although previous studies^15,16,54^ limited rapid growth measures to the first months of life and performed unadjusted correlations, this study shows associations with rapid growth in larger time frames, up to 2 years adjusting for multiple potential confounders, highlighting the robustness of these results.

The underlying mechanisms of these associations remain unclear. However, it has been speculated that the leptin surge at the end of the third trimester in humans might play a role in the development of neuronal circuits of appetite regulation.^12^ Rodent studies^55,56,57^ suggest that an early-life surge in leptin levels is crucial for the development of neural circuits in the hypothalamus, particularly the arcuate nucleus, which regulates energy balance and appetite. Although it is challenging to directly extrapolate these findings to humans due to different timing of neuronal development^58^ (occurring in the last trimester of pregnancy in humans vs the first weeks post partum in rodents^55,59^), it is notable that human leptin levels also increase during the last trimester, analogous to the postnatal leptin surge in rodents.^58,60^ If similar leptin-induced hypothalamic innervation occurs in humans, it could be speculated that elevated cord blood leptin levels are needed for the adequate development of hypothalamic neural circuits involved in energy regulation, potentially predisposing individuals with lower cord blood levels to altered metabolic programming and a higher obesity risk later in life. This theory aligns with observed negative associations between cord blood leptin levels and rapid growth. However, further research in humans is needed to verify this hypothesis.

### PYY, PP, and GLP-1

Besides leptin, this study also investigated cord blood PYY, PP, and GLP-1. Although both cord blood PYY and PP were positively associated with BMI *z* scores, only PP showed a significant association with waist to height ratio. Interestingly, in contrast to leptin, both PP and PYY levels were positively associated with rapid growth and inversely related to slow growth, despite all 3 hormones promoting satiety in adults. Exploring the precise biological mechanisms behind these associations might elucidate this unexpected finding.

The interpretation of these findings is challenging due to the limited research available on these hormones in cord blood. Previous work^10^ has suggested that high PYY levels in early postnatal life may play a role in the adaptation to enteral feeding by slowing gastric emptying and increasing small bowel transit time. This mechanism could extend the time for nutrient absorption,^10^ potentially contributing to faster growth. Unlike cord blood PYY levels, which are relatively high, cord blood PP levels are generally low compared with adult levels, although they also experience a postnatal surge in the days after birth.^9^ Unlike PYY, this postnatal increase seems not to be due to the switch to enteral feeding because PP levels also increased in preterm neonates who did not receive enteral feeds.^9^ Further research on early-life gut hormone levels is essential to uncover the molecular mechanisms underlying these observed associations.

### Strengths and Limitations

This study has several strengths. The study explored associations between a set of cord blood appetite-regulating hormones and postnatal child growth and childhood adiposity measures. The study combined multiple anthropometric measurements, including rapid growth measurements assessed over different time frames. The robustness of the findings is reinforced by their persistence after adjusting for various potential confounders and covariates.

Some study limitations should also be acknowledged. First, maternal characteristics (eg, appetite hormone levels and diet during pregnancy) and paternal BMI were not available; these factors could act as residual confounders in the observed associations. In contrast, other missing early-life factors, such as breastfeeding duration and early feeding practices, could act as effect modifiers, potentially influencing the strength or direction of associations. A second limitation of this study is the exclusion of elective cesarean sections at baseline, which may potentially have introduced collider bias. Third, our population’s low prevalence of overweight and obesity may have underestimated the strength of associations. Fourth, the study population varied slightly per hormone because not all hormones could be measured in the cord blood of all participants. Fifth, although our follow-up sample is generally representative of the ENVIRONAGE cohort, mothers with higher education levels are slightly overrepresented. Because maternal education influences child growth through socioeconomic and environmental factors, this may lead to somewhat more favorable growth trajectories in our sample, limiting generalizability to more diverse populations.

## Conclusions

In this prospective cohort study of 325 children, cord blood levels of PYY, PP, and leptin were associated with early-life growth trajectories and childhood anthropometric measurements. These results suggest that cord blood appetite hormone levels might play a role in the early-life development of overweight and obesity.

## Notes

Supplement 1. **eMethods 1.** Covariates **eMethods 2.** Appetite hormone measurements **eMethods 3.** BMI growth trajectories **eMethods 4.** Childhood adiposity measures **eTable 1.** Comparison characteristics of ENVIRONAGE participants included in this study with excluded ENVIRONAGE participants **eTable 2.** Unadjusted regression estimates between CB appetite hormones, confounders and outcomes **eTable 3.** Associations between cord blood appetite hormone levels and adiposity measures at the age of 4-6 years old, results from sensitivity analysis corrected for age and sex adjusted birthweight z scores, and interaction term with birthweight category (SGA, AGA, LGA) **eTable 4.** Associations between cord blood appetite hormone levels and body composition measures at the age of 4-6 years old, results from sensitivity analysis excluding children born to mothers with pre-eclampsia or gestational diabetes **eTable 5.** Associations between cord blood appetite hormone levels and the predicted BMI at 13 months old, results from sensitivity analysis corrected for age and sex adjusted birthweight z scores, and interaction term with birthweight category (SGA, AGA, LGA) **eTable 6.** Associations between cord blood appetite hormone levels and predicted BMI at adiposity peak, results from sensitivity analysis excluding children born to mothers with pre-eclampsia or gestational diabetes **eTable 7.** Associations between cord blood appetite hormones and postnatal growth **eTable 8.** Associations between cord blood appetite hormones and postnatal growth, excluding participants born to mothers with gestational diabetes or pre-eclampsia **eTable 9.** Associations between cord blood appetite hormones and postnatal growth, additionally correcting for birth weight **eTable 10.** Sex-specific estimates and interaction p-values from sensitivity analyses of associations between hormone levels and rapid growth **eTable 11.** Results of the spline-based mixed models for BMI growth trajectories and cord blood appetite hormones **eFigure 1.** Predicted BMI^6^ in function of the age in months with the mean (blue) and median (red) values (left) and exact median predicted BMI values per month (right) **eFigure 2.** Spearman rank correlation coefficients between the cord blood appetite hormone levels **eFigure 3.** Predicted BMI trajectories by appetite hormone levels using spline-based mixed models

Supplement 2.
Data Sharing Statement
