# Supplementary material for: Cord Blood Appetite Hormones and Early-Life Growth and Childhood Adiposity in the ENVIRONAGE Cohort
Source: JAMA Netw Open. 2025 Nov 6;8(11):e2542140. doi: 10.1001/jamanetworkopen.2025.42140 (PMC12593129; doi:10.1001/jamanetworkopen.2025.42140)
Supplement: Supplement 1. — eMethods 1. Covariates eMethods 2. Appetite hormone measurements eMethods 3. BMI growth trajectories eMethods 4. Childhood adiposity measures eTable 1. Comparison characteristics of ENVIRONAGE participants included in this study with excluded ENVIRONAGE participants eTable 2. Unadjusted regression estimates between CB appetite hormones, confounders and outcomes eTable 3. Associations between cord blood appetite hormone levels and adiposity measures at the age of 4-6 years old, results from sensitivity analysis corrected for age and sex adjusted birthweight z scores, and interaction term with birthweight category (SGA, AGA, LGA) eTable 4. Associations between cord blood appetite hormone levels and body composition measures at the age of 4-6 years old, results from sensitivity analysis excluding children born to mothers with pre-eclampsia or gestational diabetes eTable 5. Associations between cord blood appetite hormone levels and the predicted BMI at 13 months old, results from sensitivity analysis corrected for age and sex adjusted birthweight z scores, and interaction term with birthweight category (SGA, AGA, LGA) eTable 6. Associations between cord blood appetite hormone levels and predicted BMI at adiposity peak, results from sensitivity analysis excluding children born to mothers with pre-eclampsia or gestational diabetes eTable 7. Associations between cord blood appetite hormones and postnatal growth eTable 8. Associations between cord blood appetite hormones and postnatal growth, excluding participants born to mothers with gestational diabetes or pre-eclampsia eTable 9. Associations between cord blood appetite hormones and postnatal growth, additionally correcting for birth weight eTable 10. Sex-specific estimates and interaction p-values from sensitivity analyses of associations between hormone levels and rapid growth eTable 11. Results of the spline-based mixed models for BMI growth trajectories and cord blood appetite hormones eFigure 1. Predicted BMI6 in f [file jamanetwopen-e2542140-s001.pdf]

Supplemental Online Content

De Ruyter T, Michels N, Alfano R, et al. Cord blood appetite hormones and early-life growth and childhood adiposity in the ENVIRONAGE cohort. *JAMA Netw Open*. 2025;8(11):e2542140. doi:10.1001/jamanetworkopen.2025.42140

eMethods 1. Covariates
eMethods 2. Appetite hormone measurements
eMethods 3. BMI growth trajectories
eMethods 4. Childhood adiposity measures
eTable 1. Comparison characteristics of ENVIRONAGE participants included in this study with excluded ENVIRONAGE participants
eTable 2. Unadjusted regression estimates between CB appetite hormones, confounders and outcomes
eTable 3. Associations between cord blood appetite hormone levels and adiposity measures at the age of 4-6 years old, results from sensitivity analysis corrected for age and sex adjusted birthweight z scores, and interaction term with birthweight category (SGA, AGA, LGA)
eTable 4. Associations between cord blood appetite hormone levels and body composition measures at the age of 4-6 years old, results from sensitivity analysis excluding children born to mothers with pre-eclampsia or gestational diabetes
eTable 5. Associations between cord blood appetite hormone levels and the predicted BMI at 13 months old, results from sensitivity analysis corrected for age and sex adjusted birthweight z scores, and interaction term with birthweight category (SGA, AGA, LGA)
eTable 6. Associations between cord blood appetite hormone levels and predicted BMI at adiposity peak, results from sensitivity analysis excluding children born to mothers with pre-eclampsia or gestational diabetes
eTable 7. Associations between cord blood appetite hormones and postnatal growth
eTable 8. Associations between cord blood appetite hormones and postnatal growth, excluding participants born to mothers with gestational diabetes or pre-eclampsia
eTable 9. Associations between cord blood appetite hormones and postnatal growth, additionally correcting for birth weight
eTable 10. Sex-specific estimates and interaction p-values from sensitivity analyses of associations between hormone levels and rapid growth
eTable 11. Results of the spline-based mixed models for BMI growth trajectories and cord blood appetite hormones
eFigure 1. Predicted BMI[6] in function of the age in months with the mean (blue) and median (red) values (left) and exact median predicted BMI values per month (right)
eFigure 2. Spearman rank correlation coefficients between the cord blood appetite hormone levels
eFigure 3. Predicted BMI trajectories by appetite hormone levels using spline-based mixed models

This supplemental material has been provided by the authors to give readers additional information about their work.

© 2025 De Ruyter et al. *JAMA Netw Open.*

eMethods 1. Covariates

After delivery, the mothers completed questionnaires, providing detailed lifestyle and sociodemographic information about the mother (e.g., maternal age and education level, parity, smoking habits and alcohol use during pregnancy). Further information on the delivery and health status of the mother and newborn was derived from medical records of the hospital (e.g., newborn sex, mode of delivery, gestational age at delivery, birth weight, birth length, pre-pregnancy weight and height, gestational weight gain and pregnancy complications i.e. gestational diabetes and preeclampsia). Parity was categorized as mothers having their first, second, third or more child. Maternal education was categorized by International Standard Classification of Education[1] and coded as "low," no diploma, primary school or lower secundary (1st - 3th middle school); "middle", high school diploma; or "high", college or university degree. Smoking and alcohol use during pregnancy were binary coded (yes/no). Gestational age was based on the date of conception that was estimated based on maternity records of the last menstrual period, or, if not available, it was based on the initial ultrasonographic examination. Maternal height and weight were measured without shoes, wearing light clothes to the nearest centimeter and weight to the nearest 0.1 kg at the first antenatal visit of each pregnancy (weeks 7–9 of gestation). BMI was defined as weight in kilograms divided by the square of height in meters. Breastfeeding (yes/no) was reported by the mother at the first follow-up visit.

eMethods 2 - Appetite hormone measurements

Immediately after delivery, CB was collected in BD Vacutainer® plastic tubes spray-coated with K2EDTA (BD, Franklin Lakes, NJ, USA). Within a 20-minute window from collection, the tubes underwent centrifugation at 3,200 rpm for 15 minutes after which the plasma was stored at −80°C until further analysis. PYY and GLP-1 levels were measured using previously described radioimmunoassays[2,3] (RIA) where all samples were run in one batch. Intra-assay coefficients of variation (CV) were 2.99% and 4.01% for PYY and GLP-1 assays, respectively.

Leptin and PP levels were measured using EMD Millipore MILLIPLEX Human Metabolic Hormone Magnetic Bead Panel (HMH3-34K, Millipore, Merck, Darmstadt, Germany), according to the manufacturer's instructions. The inter-assay CVs were 11.36% and 43.58% and intra-assay CVs were 7.87% and 31.76%, for leptin and PP measurements, respectively. Measurements were performed using the Bio-Plex MAGPIX multiplex reader, and data were analyzed using the Bio-Plex Manager 6.1 software (Bio-Rad). To account for batch effects, the PP and leptin levels were normalized across batches, and these normalized values were used in all following statistical analyses.

© 2025 De Ruyter et al. *JAMA Netw Open.*

eMethods 3 - BMI growth trajectories

BMI growth trajectories from birth up to 29 months were modeled using natural cubic splines of age in days (df=4), with internal knots placed at 24, 117, and 407 days (~ 3 weeks, 4 and 13.5 months) to capture non-linear growth. Associations between cord blood appetite hormone levels and BMI growth trajectories were analyzed using linear mixed-effects models incorporating natural cubic splines of age as described above and random intercept of the subject and random intercept and slopes on the spline using R package lme4. Hormone levels were included as interaction terms with spline-based age to assess their influence on BMI trajectories.

eMethods 4 Childhood adiposity measures

Weight and height were measured bare foot and lightly clothed, using a Body Composition Monitor (KaradaScan, OMRON) and a fixed stadiometer to the nearest 0.1%, 0.1kg and 0.5 cm, respectively. BMI was calculated as the ratio of weight (kg) over squared height (m^2). Age-adjusted BMI, length and weight z-scores were calculated according to the World Health Organization's (WHO) Child Growth Standards using the R package 'anthro'.[4] Waist circumference was measured at the level of the umbilicus to the nearest 0.1 cm. WHtR was calculated as the ratio of waist circumference (cm) over height (cm).

© 2025 De Ruyter et al. *JAMA Netw Open.*

1. eTABLES

eTable1 - Comparison characteristics of ENVIRONAGE participants included in this study with excluded ENVIRONAGE participants

**Characteristic**	**N**	**Included ENVIR*ON*AGE participants** N = 325	**Excluded ENVIR*ON*AGE participants**, N = 1,656	**p-value**[a]
Newborn				
Sex	1,981			0.14
Male		151 (46%)	846 (51%)	
Female		174 (54%)	810 (49%)	
Birthweight, g	1,981	3,390.00 ± 650.00	3,415.00 ± 615.00	0.4
Gestation duration, weeks	1,981	39.00 ± 2.00	39.00 ± 2.00	0.6
Maternal				
Age at delivery, years	1,981	30.00 ± 6.00	29.00 ± 5.00	0.056
Gestational weight gain, kg	1,924	14.00 ± 6.15	14.00 ± 7.50	0.7
Pre-pregnancy BMI, kg/m^2	1,953	23.50 ± 4.90	24.00 ± 6.40	0.084
Parity	1,981			0.065
First child		180 (55%)	878 (53%)	
Second child		116 (36%)	551 (33%)	
Third or more child		29 (8.9%)	226 (14%)	
Education level[b]	1,868			<0.001
Low		25 (7.7%)	175 (11%)	
Middle		91 (28%)	575 (37%)	
High		209 (64%)	793 (51%)	
Gestational diabetes	1,979	13 (4.0%)	87 (5.3%)	0.4
Preeclampsia	1,979	3 (0.9%)	18 (1.1%)	>0.9
Alcohol use during pregnancy	1,869	65 (20%)	179 (12%)	<0.001
Breastfeeding	449	215 (74%)	116 (73%)	0.7

© 2025 De Ruyter et al. *JAMA Netw Open.*

Characteristic	N	Included ENVIR*ON*AGE participants N = 325	Excluded ENVIR*ON*AGE participants, N = 1,656	p-value[a]
Smoking during pregnancy	1,881	37 (11%)	176 (11%)	>0.9
Predicted BMI at adiposity peak, kg/m²	643	17.31 ± 1.66	17.36 ± 1.45	0.3
Growth the first 6 months	644			0.7
slow growth		105 (32%)	103 (32%)	
normal growth		176 (54%)	165 (52%)	
rapid growth		44 (14%)	51 (16%)	
Growth the first year	644			0.9
slow growth		63 (19%)	57 (18%)	
normal growth		159 (49%)	157 (49%)	
rapid growth		103 (32%)	105 (33%)	
Growth the first 2 years	644			0.4
slow growth		49 (15%)	39 (12%)	
normal growth		127 (39%)	139 (44%)	
rapid growth		149 (46%)	141 (44%)	
BMI z-score	697	0.50 ± 1.11	0.44 ± 1.12	0.6
Waist-height ratio	694	0.49 ± 0.04	0.49 ± 0.05	0.11
Overweight[d]	698	44 (14%)	54 (14%)	0.8
Obesity[d]	698	7 (2.2%)	10 (2.7%)	0.8

[a] Pearson's Chi-squared test; Wilcoxon rank sum test

[b]Maternal education was categorized by International Standard Classification of Education[1] and coded as "low," no diploma, primary school or lower secundary (1st - 3th middle school); "middle", high school diploma; or "high", college or university degree.

[c]BMI z-scores were calculated according to the World Health Organization's (WHO) Child Growth Standards based on length/height, weight, and age of the child.

[d] Overweight and obesity were determined based on the cut-offs proposed by Cole et al. using BMI z-score

© 2025 De Ruyter et al. *JAMA Netw Open.*

eTable 2 - Unadjusted regression estimates between CB appetite hormones, confounders and outcomes.

	GLP-1			PYY			PP			Leptin		
	Estimate	p-value	R^2	Estimate	p-value	R^2	Estimate	p-value	R^2	Estimate	p-value	R^2
Newborn characteristics												
Sex, male as reference	0.012	0.79	0.0003	0.056	0.38	0.0033	-0.059	0.64	0.0010	0.335	0.01	0.0337
Birth weight, g	0.000	0.12	0.0107	0.000	0.03	0.0205	0.000	0.33	0.0042	0.001	<0.001	0.1809
Gestational age, weeks	-0.006	0.69	0.0007	0.026	0.23	0.0063	0.011	0.76	0.0004	0.160	<0.001	0.0882
Maternal characteristics												
Pre-pregnancy BMI	0.001	0.87	0.0001	0.007	0.35	0.0038	0.021	0.14	0.0098	0.019	0.17	0.0085
Gestational weight gain, kg	-0.006	0.14	0.0097	-0.013	0.03	0.0216	-0.011	0.34	0.0042	0.023	0.04	0.0197
Maternal age at delivery, years	-0.011	0.03	0.0215	-0.008	0.31	0.0044	0.009	0.50	0.0020	0.023	0.08	0.0133
Parity, first child as reference												
second child	-0.101	0.03	0.0255	-0.171	0.01	0.0394	-0.051	0.70	0.0098	-0.004	0.97	0.0090
third or more child	-0.117	0.14	0.0255	-0.258	0.03	0.0394	-0.324	0.14	0.0098	-0.298	0.17	0.0090
Method of delivery, vaginal birth as reference	-0.192	0.16	0.0088	0.040	0.84	0.0002	-0.121	0.69	0.0007	0.055	0.85	0.0002
Maternal education, low as reference												
medium	0.039	0.69	0.0009	0.193	0.18	0.0085	0.115	0.63	0.0017	-0.038	0.87	0.0164
high	0.042	0.65	0.0009	0.180	0.18	0.0085	0.036	0.87	0.0017	0.215	0.32	0.0164
Smoking during pregnancy	0.011	0.87	0.0001	-0.031	0.76	0.0004	-0.130	0.50	0.0020	-0.182	0.34	0.0041
Alcohol use during pregnancy	-0.120	0.03	0.0202	0.017	0.83	0.0002	0.206	0.17	0.0084	0.048	0.75	0.0005
Breastfeeding	-0.026	0.63	0.0011	-0.084	0.28	0.0057	0.080	0.59	0.0015	-0.004	0.98	0.0000
Child's characteristics at follow-up												
Rapid growth first six months	0.017	0.79	0.0003	0.177	0.06	0.0155	0.206	0.25	0.0059	-0.370	0.04	0.0196
Rapid growth in the first year	0.003	0.95	0.0000	0.140	0.05	0.0174	0.268	0.04	0.0183	-0.473	<0.001	0.0587
Rapid growth in the first two years	0.034	0.43	0.0027	0.158	0.01	0.0268	0.354	0.00	0.0365	-0.565	<0.001	0.0957
Predicted BMI at 9 months old	-0.004	0.81	0.0003	0.026	0.34	0.0041	0.064	0.21	0.0069	0.041	0.42	0.0029
Zbmi score	-0.003	0.92	0.0000	0.054	0.18	0.0080	0.097	0.18	0.0079	-0.047	0.51	0.0020
Waist-to-height ratio	-0.433	0.55	0.0016	0.817	0.45	0.0026	2.727	0.19	0.0076	1.193	0.56	0.0015

Abbreviations: GLP-1, Glucagon-like-peptide-1; PYY, Peptide tyrosine tyrosine; PP, Pancreatic polypeptide; CB, cord blood; BMI, Body Mass Index

eTable 3 - Associations between cord blood appetite hormone levels and adiposity measures at the age of 4-6 years old, , results from sensitivity analysis corrected for age and sex adjusted birthweight z scores, and interaction term with birthweight category (SGA, AGA, LGA)

	N	Basic models[a]		Fully adjusted models[b]	
		β* (95% CI)[c]	*p-value*	β* (95% CI)[c]	*p-value*
Waist-to-height ratio					
GLP-1	229	-0.08 (-0.22 to 0.05)	0.20	-0.08 (-0.23 to 0.06)	0.28
PYY	229	0.07 (-0.06 to 0.20)	0.29	0.07 (-0.08 to 0.22)	0.34
PP	226	0.16 (0.03 to 0.28)	0.02	0.13 (-0.01 to 0.28)	0.07
Leptin	226	-0.02 (-0.16 to 0.12)	0.76	0.02 (-0.14 to 0.19)	0.79
BMI z-score					
GLP-1	229	-0.012 (-0.15 to 0.12)	0.86	-0.02 (-0.17 to 0.12)	0.78
PYY	229	0.15 (-0.03 to 0.29)	0.03	0.18 (0.05 to 0.30)	0.007
PP	226	0.13 (-0.006 to 0.27)	0.06	0.16 (0.02 to 0.29)	0.02
Leptin	226	-0.11 (-0.25 to 0.04)	0.14	-0.04 (-0.19 to 0.10)	0.55

[a]Basic models were adjusted for sex, age at follow-up visit, birthweight z-score and birthweight category (SGA, AGA, LGA)
[b]Fully adjusted models were further adjusted for breastfeeding, gestational age, mode of delivery, maternal smoking and alcohol use during pregnancy, maternal education, maternal age at delivery, gestational weight gain, pre-pregnancy BMI, and parity.
[c] Estimates are presented as standardized regression coefficients.

Abbreviations: GLP-1, Glucagon-like-peptide-1; PYY; Peptide tyrosine tyrosine, PP; Pancreatic polypeptide; BMI, Body Mass Index; 95%CI, 95% Confidence interval; SGA, small for gestational age; AGA, appropriate for gestational age; LGA, large for gestational age.

eTable 4 - Associations between cord blood appetite hormone levels and body composition measures at the age of 4-6 years old, results from sensitivity analysis excluding children born to mothers with pre-eclampsia or gestational diabetes.

	N	Basic models		Fully adjusted models	
		β* (95% CI)	*p*-value	β* (95% CI)	*p*-value
Waist-to-height ratio					
GLP-1	219	-0.08(-0.21 to 0.05)	0.24	-0.09 (-0.23, 0.06)	0.24
PYY	219	0.06 (-0.07 to 0.19)	0.38	0.08 (-0.07, 0.22)	0.31
PP	213	0.14 (0.01, 0.27)	0.03	0.12 (-0.02, 0.27)	0.09
Leptin	213	-0.03 (-0.17, 0.11)	0.65	0.01 (-0.14, 0.16)	0.87
BMI z-score					
GLP-1	219	-0.02 (-0.15, 0.12)	0.80	-0.02 (-0.15, 0.12)	0.81
PYY	219	0.11 (-0.03, 0.24)	0.12	0.16 (0.03, 0.30)	0.01
PP	213	0.13 (-0.02, 0.27)	0.08	0.17 (0.03, 0.31)	0.01
Leptin	213	-0.07 (-0.21, 0.07)	0.33	-0.004 (-0.180, 0.16)	0.96

Estimates are presented as standardized regression coefficients. Basic models were adjusted for sex andage at follow-up visit. Fully adjusted models were additionally adjusted for breastfeeding, gestational age, mode of delivery, maternal smoking and alcohol use during pregnancy, maternal education, maternal age at delivery, gestational weight gain, pre-pregnancy BMI, and parity. Abbreviations: : GLP-1, Glucagon-like-peptide-1; PYY; Peptide tyrosine tyrosine, PP; Pancreatic polypeptide; BMI, Body Mass Index; 95%CI, 95% Confidence interval;

© 2025 De Ruyter et al. *JAMA Netw Open.*

eTable 5 - Associations between cord blood appetite hormone levels and the predicted BMI at 13 months old, results from sensitivity analysis corrected for age and sex adjusted birthweight z scores, and interaction term with birthweight category (SGA, AGA, LGA)

	N	Basic models[a]		Fully adjusted models[b]	
		β* (95% CI)[c]	*p*-value	β* (95% CI)[c]	*p*-value
GLP-1	229	-0.01 (-0.14 to 0.13)	0.94	0.02 (-0.13 to 0.12)	0.97
PYY	229	0.13 (-0.004 to 0.26)	0.06	0.18 (0.06 to 0.31)	0.005
PP	226	0.09(-0.03 to 0.22)	0.15	0.11 (-0.02 to 0.24)	0.11
Leptin	226	-0.06 (-0.20 to 0.07)	0.37	-0.04 (-0.20 to 0.11)	0.58

[a]Basic models were adjusted for sex, birthweight and birthweight category (SGA,AGA, LGA).

[b] Fully adjusted models were further adjusted for breastfeeding, gestational age, mode of delivery, maternal smoking and alcohol use during pregnancy, maternal education, maternal age at delivery, gestational weight gain, pre-pregnancy BMI, and parity.

[C] Estimates are presented as standardized regression coefficients.

Abbreviations: GLP-1, Glucagon-like-peptide-1; PYY; Peptide tyrosine tyrosine, PP; Pancreatic polypeptide; BMI, Body Mass Index; 95%CI, 95% Confidence interval; SGA, small for gestational age; AGA, appropriate for gestational age; LGA, large for gestational age.

eTable 6 - Associations between cord blood appetite hormone levels and predicted BMI at adiposity peak, results from sensitivity analysis excluding children born to mothers with pre-eclampsia or gestational diabetes.

	N	Basic models		Fully adjusted models	
		β* (95% CI)	*p*-value	β* (95% CI)	*p*-value
GLP-1	219	-0.03 (-0.17 to 0.10)	0.64	0.001 (-0.17 to 0.17)	0.99
PYY	219	0.05 (-0.09 to 0.19)	0.49	0.14 (-0.01 to 0.29)	0.06
PP	213	0.08 (-0.05 to 0.22)	0.24	0.12 (-0.02 to 0.26)	0.09
Leptin	213	0.02 (-0.11 to 0.16)	0.74	0.06 (-0.09 to 0.21)	0.40

Estimates are presented as standardized regression coefficients. Basic models were adjusted for sex and birth weight. Fully adjusted models were further adjusted for breastfeeding, gestational age, mode of delivery, maternal smoking and alcohol use, maternal education, maternal age, gestational weight gain, pre-pregnancy BMI, and parity.

Abbreviations:GLP-1, Glucagon-like-peptide-1; PYY; Peptide tyrosine tyrosine, PP; Pancreatic polypeptide; BMI, Body Mass Index; 95%CI, 95% Confidence interval;

© 2025 De Ruyter et al. *JAMA Netw Open.*

eTable 7 - Associations between cord blood appetite hormones and postnatal growth

	N	Basic models		Fully adjusted models	
		OR (95% CI)	*p*-value	OR (95% CI)	*p*-value
**Birth - 6 months**					
**GLP-1**	230				
slow growth		0.64 (0.33, 1.19)	0.15	0.78 (0.38, 1.61)	0.50
rapid growth		0.94 (0.41, 2.18)	0.88	1.09 (0.41, 2.90)	0.87
**PYY**	229				
slow growth		0.58 (0.38, 0.89)	0.01	0.44 (0.26, 0.75)	0.003
rapid growth		1.48 (0.78, 2.84)	0.23	2.88 (1.25, 6.64)	0.01
**PP**	226				
slow growth		0.81 (0.64, 1.03)	0.09	0.72 (0.56, 0.94)	0.02
rapid growth		1.15 (0.79, 1.68)	0.47	1.23 (0.78, 1.95)	0.38
**Leptin**	226				
slow growth		2.68 (1.82, 3.92)	<0.001	2.90 (1.81, 4.63)	<0.001
rapid growth		0.91 (0.7, 1.18)	0.47	1.1 (0.8, 1.51)	0.54
**Birth - 12 months**					
**GLP-1**	230				
slow growth		0.43 (0.19, 0.95)	0.04	0.52 (0.21, 1.28)	0.16
rapid growth		0.82 (0.43, 1.55)	0.53	0.80 (0.4, 1.58)	0.51
**PYY**	229				
slow growth		0.5 (0.3, 0.83)	0.007	0.4 (0.22, 0.74)	0.004
rapid growth		1.3 (0.81, 2.08)	0.28	1.76 (1, 3.1)	0.05
**PP**	226				
slow growth		1.03 (0.8, 1.34)	0.81	0.95 (0.68, 1.32)	0.75
rapid growth		1.35 (1.01, 1.79)	0.04	1.39 (1.01, 1.92)	0.05
**Leptin**	226				
slow growth		2.48 (1.57, 3.9)	<0.001	3.14 (1.73, 5.68)	<0.001
rapid growth		0.64 (0.45, 0.92)	0.02	0.61 (0.39, 0.97)	0.04
**Birth - 24 months**					
**GLP-1**	230				
slow growth		0.41 (0.16, 1.04)	0.06	0.45 (0.15, 1.36)	0.16
rapid growth		1.05 (0.58, 1.9)	0.94	0.93 (0.48, 1.82)	0.84
**PYY**	229				
slow growth		0.49 (0.28, 0.86)	0.01	0.42 (0.21, 0.83)	0.01
rapid growth		1.38 (0.9, 2.13)	0.14	1.71 (1.03, 2.84)	0.04
**PP**	226				
slow growth		1.09 (0.81, 1.47)	0.57	0.99 (0.65, 1.5)	0.96
rapid growth		1.51 (1.14, 2.01)	0.004	1.65 (1.2, 2.27)	0.002
**Leptin**	226				
slow growth		1.74 (1.06, 2.84)	0.03	2.49 (1.26, 4.94)	0.009
rapid growth		0.45 (0.31, 0.65)	<0.001	0.43 (0.27, 0.66)	<0.001

Relative Odds ratio's (normal growth as reference category) are shown for a doubling in CB hormone level. Basic models were adjusted for sex, fully adjusted models were further adjusted for maternal age at delivery, maternal education level, pre-pregnancy BMI, gestational weight gain, maternal smoking and alcohol use during pregnancy, parity, gestational age at delivery, delivery method and breastfeeding. Abbreviations:GLP-1, Glucagon-like-peptide-1; PYY, Peptide tyrosine tyrosine; PP, Pancreatic polypeptide; 95%CI, 95% Confidence ; OR, Odds ratio; CB, cord blood

© 2025 De Ruyter et al. *JAMA Netw Open.*

eTable 8 - Associations between cord blood appetite hormones and postnatal growth, excluding participants born to mothers with gestational diabetes or pre-eclampsia

		Basic models		Fully adjusted models	
	N	OR (95% CI)	*p*-value	OR (95% CI)	*p*-value
		**Birth - 6 months**			
**GLP-1**	219				
slow growth		0.62 (0.33, 1.16)	0.14	0.76 (0.37,	0.46
rapid		0.88 (0.38, 2.08)	0.78	0.96 (0.34,	0.93
**PYY**	219				
slow growth		0.56 (0.37, 0.87)	0.009	0.44 (0.26,	0.002
rapid		1.44 (0.74, 2.8)	0.28	2.96 (1.22,	0.02
**PP**	213				
slow growth		0.82 (0.65, 1.04)	0.11	0.72 (0.55,	0.02
rapid		1.15 (0.78, 1.7)	0.47	1.16 (0.71,	0.54
**Leptin**	213				
slow growth		2.69 (1.83, 3.96)	<0.001	2.84 (1.78,	<0.001
rapid		0.9 (0.69, 1.17)	0.44	1.15 (0.83, 1.6)	0.40
		**Birth - 12 months**			
**GLP-1**	219				
slow growth		0.42 (0.19, 0.93)	0.03	0.49 (0.2, 1.22)	0.12
rapid		0.78 (0.41, 1.48)	0.44	0.65 (0.3, 1.4)	0.27
**PYY**	219				
slow growth		0.49 (0.3, 0.81)	0.006	0.38 (0.21,	0.003
rapid		1.25 (0.78, 2.01)	0.36	1.71 (0.93,	0.09
**PP**	213				
slow growth		1.05 (0.81, 1.37)	0.71	0.96 (0.69,	0.82
rapid		1.37 (1.02, 1.84)	0.03	1.42 (1.01,	0.04
**Leptin**	213				
slow growth		2.42 (1.53, 3.82)	<0.001	3.04 (1.68,	<0.001
rapid		0.62 (0.43, 0.9)	0.01	0.62 (0.39, 1)	0.05
		**Birth - 24 months**			
**GLP-1**	219				
slow growth		0.41 (0.16, 1.02)	0.06	0.45 (0.15,	0.15
rapid		1.01 (0.55, 1.83)	0.98	0.87 (0.43,	0.69
**PYY**	219				
slow growth		0.47 (0.27, 0.84)	0.01	0.4 (0.2, 0.81)	0.01
rapid		1.3 (0.84, 2.01)	0.24	1.57 (0.92,	0.10
**PP**	213				
slow growth		1.1 (0.82, 1.49)	0.52	1 (0.66, 1.51)	0.99
rapid		1.53 (1.14, 2.04)	0.004	1.67 (1.2, 2.34)	0.003
**Leptin**	213				
slow growth		1.69 (1.04, 2.76)	0.04	2.42 (1.22,	0.01
rapid		0.41 (0.28, 0.62)	<0.001	0.41 (0.26,	<0.001

Relative Odds ratio's (normal growth as reference category) are shown for a doubling in CB hormone level. Basic models were adjusted for sex, fully adjusted models were further adjusted for maternal age at delivery, maternal education level, pre-pregnancy BMI, gestational weight gain, maternal smoking and alcohol use during pregnancy, parity, gestational age at delivery, delivery method and breastfeeding.

Abbreviations:GLP-1, Glucagon-like-peptide-1; PYY, Peptide tyrosine tyrosine; PP, Pancreatic polypeptide; 95%CI, 95% Confidence ; OR, Odds ratio; CB, cord blood

eTable 9 - Associations between cord blood appetite hormones and postnatal growth, additionally correcting for birth weight.

	N	Basic models		Fully adjusted models	
		OR (95% CI)	*p*-value	OR (95% CI)	*p*-value
**Birth - 6 months**					
**GLP-1**	230				
slow growth		0.67 (0.51, 0.89)	0.005	0.76 (0.68, 0.85)	<0.001
rapid growth		0.86 (0.57, 1.31)	0.49	1.15 (1.08, 1.22)	<0.001
**PYY**	229				
slow growth		0.61 (0.43, 0.87)	0.007	0.44 (0.29, 0.66)	<0.001
rapid growth		1.42 (0.96, 2.09)	0.08	2.74 (2.43, 3.09)	<0.001
**PP**	226				
slow growth		0.83 (0.66, 1.03)	0.08	0.74 (0.57, 0.95)	0.02
rapid growth		1.13 (0.8, 1.6)	0.47	1.17 (0.78, 1.75)	0.45
**Leptin**	226				
slow growth		1.86 (1.5, 2.3)	<0.001	2.3 (1.58, 3.36)	<0.001
rapid growth		1.15 (0.91, 1.45)	0.24	1.26 (0.9, 1.78)	0.18
**Birth - 12 months**					
**GLP-1**	230				
slow growth		0.44 (0.34, 0.58)	<0.001	0.49 (0.46, 0.52)	<0.001
rapid growth		0.75 (0.48, 1.15)	0.19	0.82 (0.75, 0.88)	<0.001
**PYY**	229				
slow growth		0.51 (0.33, 0.77)	0.002	0.39 (0.29, 0.53)	<0.001
rapid growth		1.22 (0.87, 1.71)	0.25	1.54 (0.97, 2.44)	0.07
**PP**	226				
slow growth		1.08 (0.81, 1.43)	0.62	1.03 (0.7, 1.5)	0.89
rapid growth		1.31 (1, 1.72)	0.05	1.35 (0.98, 1.84)	0.06
**Leptin**	226				
slow growth		1.72 (1.33, 2.21)	<0.001	2.47 (1.59, 3.84)	<0.001
rapid growth		0.9 (0.73, 1.12)	0.34	0.79 (0.54, 1.14)	0.21
**Birth - 24 months**					
**GLP-1**	230				
slow growth		0.42 (0.4, 0.45)	<0.001	0.44 (0.41, 0.46)	<0.001
rapid growth		1.05 (0.85, 1.29)	0.67	1.04 (0.98, 1.11)	0.19
**PYY**	229				
slow growth		0.51 (0.34, 0.78)	0.002	0.42 (0.38, 0.46)	<0.001
rapid growth		1.37 (0.97, 1.94)	0.07	1.65 (1.11, 2.47)	0.02
**PP**	226				
slow growth		1.11 (0.82, 1.5)	0.51	1.03 (0.68, 1.55)	0.90
rapid growth		1.5 (1.13, 1.98)	0.005	1.62 (1.18, 2.24)	0.003
**Leptin**	226				
slow growth		1.37 (1.06, 1.77)	0.02	2.4 (1.72, 3.35)	<0.001
rapid growth		0.63 (0.51, 0.78)	<0.001	0.6 (0.43, 0.84)	0.003

Relative Odds ratio's (normal growth as reference category) are shown for a doubling in CB hormone level. Basic models were adjusted for sex, fully adjusted models were further adjusted for maternal age at delivery, maternal education level, pre-pregnancy BMI, gestational weight gain, maternal smoking and alcohol use during pregnancy, parity, gestational age at delivery, delivery method and breastfeeding. Abbreviations:GLP-1, Glucagon-like-peptide-1; PYY, Peptide tyrosine tyrosine; PP, Pancreatic polypeptide; 95%CI, 95% Confidence ; OR, Odds ratio; CB, cord blood

© 2025 De Ruyter et al. *JAMA Netw Open.*

eTable 10 - Sex-specific estimates and interaction p-values from sensitivity analyses of associations between hormone levels and rapid growth.

	p-interaction Hormone*Sex	Girls		Boys	
		OR (95% CI)	*p*-value	OR (95% CI)	*p*-value
**Birth – 6 months**					
**GLP-1**					
slow growth	>0.99	0.76 (0.69, 0.82)	<0.001	0.65 (0.54, 0.78)	<0.001
rapid growth	<0.001	0.9 (0.74, 1.08)	0.247	2.06 (1.39, 3.07)	0.015
**PYY**					
slow growth	0.01	0.53 (0.29,0.98)	0.044	0.38(0.21, 0.69)	0.002
rapid growth	<0.001	2.82 (2.08, 3.81)	<0.001	1.32(0.69, 2.53)	0.181
**PP**					
slow growth	<0.001	0.73 (0.52, 1.02)	0.062	0.85(0.52, 1.39)	0.50
rapid growth	0.609	1.16 (0.59, 2,28)	0.661	1.26(0.74, 2.14)	0.39
**Leptin**					
slow growth	<0.001	1.33 (0.7, 2.52)	0.38	3.54(1.88, 6.64)	<0.001
rapid growth	<0.001	0.79 (0.55, 1.13)	0.66	1.59 (0.88, 2.87)	0.125
**Birth – 12 months**					
**GLP-1**					
slow growth	<0.001	0.77 (0.74, 0.81)	<0.001	0.28 (0.24, 0.34)	<0.001
rapid growth	<0.001	0.7 (0.63, 0.77)	<0.001	1.32 (1.18, 1.48)	<0.001
**PYY**					
slow growth	<0.001	0.72 (0.68, 0.77)	<0.001	0.25 (0.15, 0.43)	<0.001
rapid growth	0.01	1.8 (0.95,3.44)	0.073	1.46 (0.73, 2.9)	0.28
**PP**					
slow growth	0.91	1.48 (0.85,2.55)	0.16	0.97 (0.52, 1.81)	0.92
rapid growth	<0.001	1.72 (1.1,2.94)	0.046	1.23 (0.78, 1.92)	0.37
**Leptin**					
slow growth	<0.001	1.82 (1.03, 3.23)	0.039	3.01 (1.4, 6.47)	0.006
rapid growth	0.03	0.82 (0.45,1.51)	0.52	0.75 (0.45, 1.25)	0.27
**Birth – 24 months**					
**GLP-1**					
slow growth	<0.001	0.32 (0.31, 0.33)	<0.001	0.89 (0.65, 1.2)	0.373
rapid growth	<0.001	0.72 (0.65, 0.81)	<0.001	2.52 (2.07, 3.07)	<0.001
**PYY**					
slow growth	0.17	0.47 (0.44, 0.5)	<0.001	0.48 (0.39, 0.59)	<0.001
rapid growth	<0.001	1.53 (0.82, 2.86)	0.18	2.13 (1.21, 3.75)	0.01
**PP**					
slow growth	0.86	1.35 (0.77, 2.35)	0.29	1.05 (0.51, 2.16)	0.90
rapid growth	<0.001	2.01 (1.21, 3.32)	0.007	1.53 (0.97, 2.41)	0.07
**Leptin**					
slow growth	0.062	2.45 (1.98, 3.04)	<0.001	2.66 (0.88, 8.06)	0.07
rapid growth	<0.001	0.96 (0.55, 1.67)	0.873	0.38 (0.22, 0.64)	<0.001

Relative Odds ratio's (normal growth as reference category) are shown for a doubling in CB hormone level. Models were adjusted for for maternal age at delivery, maternal education level, pre-pregnancy BMI, gestational weight gain, maternal smoking and alcohol use during pregnancy, parity, gestational age at delivery, delivery method and breastfeeding. Abbreviations:GLP-1, Glucagon-like-peptide-1; PYY, Peptide tyrosine tyrosine; PP, Pancreatic polypeptide; 95%CI, 95% Confidence ; OR, Odds ratio; CB, cord blood

© 2025 De Ruyter et al. *JAMA Netw Open.*

eTable 11 - Results of the spline-based mixed models for BMI growth trajectories and cord blood appetite hormones

Fixed effects	Estimate (95% CI)	P value
**GLP-1**		
Intercept	13.87 (12.06, 15.67)	<0.001
Sex – Female	−0.10 (−0.33, 0.13)	0.401
Splines 1	4.04 (1.54, 6.54)	0.002
Splines 2	0.37 (−3.09, 3.83)	0.835
Splines 3	3.70 (−0.00, 7.40)	0.051
Splines 4	1.66 (−2.05, 5.36)	0.381
Log transformed GLP-1	−0.13 (−0.63, 0.37)	0.601
Splines 1 * Log GLP-1	0.11 (−0.58, 0.80)	0.763
splines2 * Log GLP-1	0.08 (−0.88, 1.04)	0.877
Splines 3 * Log GLP-1	0.32 (−0.70, 1.35)	0.537
Splines 4 * Log GLP-1	0.15 (−0.88, 1.19)	0.774
**Leptin**		
Intercept	6.91 (4.65, 9.17)	<0.001
Sex – Female	−0.15 (−0.39, 0.09)	0.224
Splines 1	11.04 (7.80, 14.28)	<0.001
Splines 2	3.68 (−0.99, 8.34)	0.125
Splines 3	15.46 (10.61, 20.31)	<0.001
Splines 4	8.18 (3.43, 12.92)	0.001
Log transformed Leptin	0.65 (0.42, 0.89)	<0.001
Splines 1 * Log Leptin	−0.66 (−0.99, −0.33)	<0.001
splines2 * Log Leptin	−0.31 (−0.78, 0.17)	0.204
Splines 3 * Log Leptin	−1.08 (−1.57, −0.58)	<0.001
Splines 4 * Log Leptin	−0.60 (−1.09, −0.12)	0.016
**PYY**		
Intercept	14.52 (12.93 to 16.11)	<0.001
Sex – Female	-0.10 (-0.33 to 0.13)	0.408
Splines 1	3.14 (0.94 to 5.35)	0.006
Splines 2	-1.50 (-4.57 to 1.58)	0.341
Splines 3	0.60 (-2.66 to 3.86)	0.719
Splines 4	-0.21 (-3.48 to 3.06)	0.900
Log transformed PYY	-0.24 (-0.57 to 0.09)	0.157
Splines 1 * Log PYY	0.27 (-0.19 to 0.74)	0.255
splines2 * Log PYY	0.46 (-0.19 to 1.11)	0.170
Splines 3 * Log PYY	0.90 (0.22 to 1.59)	0.010 *
Splines 4 * Log PYY	0.51 (-0.18 to 1.21)	0.150
**PP**		
Intercept	13.94 (13.04 to 14.83)	<0.001
Sex – Female	-0.10 (-0.34 to 0.15)	0.437
Splines 1	3.15 (1.93 to 4.37)	<0.001
Splines 2	0.75 (-1.00 to 2.50)	0.403
Splines 3	3.01 (1.19 to 4.83)	0.001
Splines 4	1.47 (-0.33 to 3.27)	0.112
Log transformed PP	-0.18 (-0.43 to 0.07)	0.162
Splines 1 * Log PP	0.39 (0.05 to 0.74)	0.026
splines2 * Log PP	-0.03 (-0.53 to 0.46)	0.896
Splines 3 * Log PP	0.54 (0.03 to 1.06)	0.040
Splines 4 * Log PP	0.23 (-0.28 to 0.75)	0.372

© 2025 De Ruyter et al. *JAMA Netw Open.*

Abbreviations:GLP-1, Glucagon-like-peptide-1; PYY, Peptide tyrosine tyrosine; PP, Pancreatic polypeptide; 95%CI, Confidence ;

2. SUPPLEMENTARY FIGURES

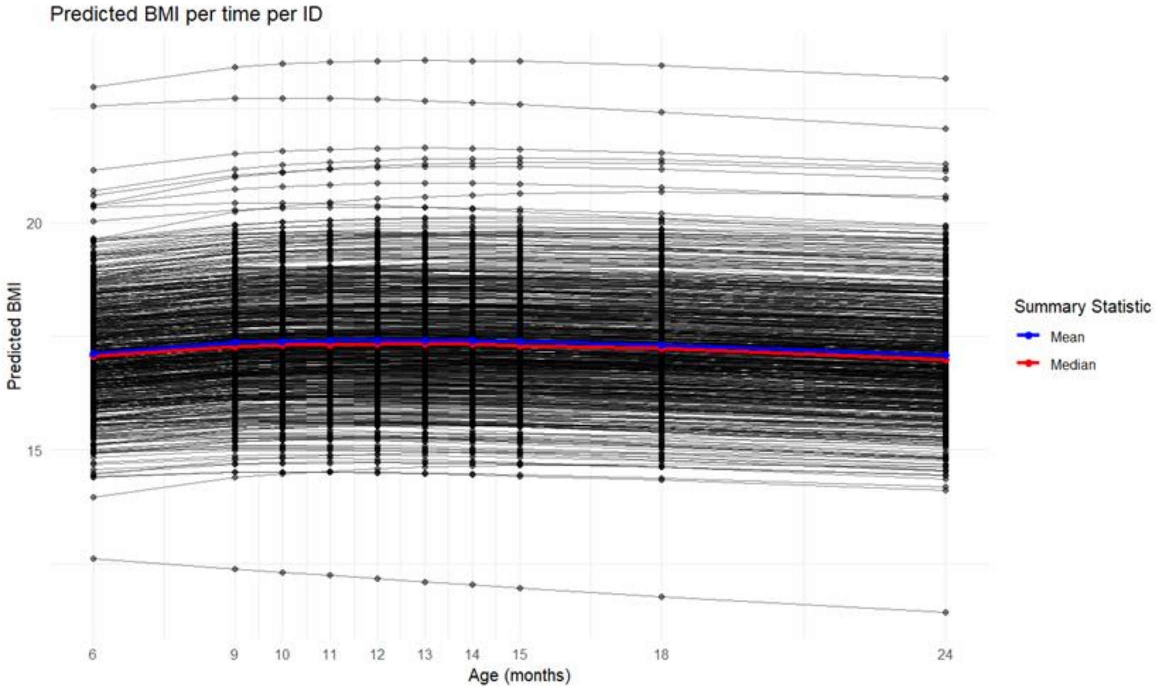

Age (Months)	Median predicted BMI
6	17.058
9	17.260
10	17.299
11	17.316
12	17.327
13	17.330
14	17.318
15	17.298
18	17.225
24	16.985

eFigure 1 – Predicted BMI.[5] in function of the age in months with the mean (blue) and median (red) values (left) and exact median predicted BMI values per month (right).

© 2025 De Ruyter et al. *JAMA Netw Open.*

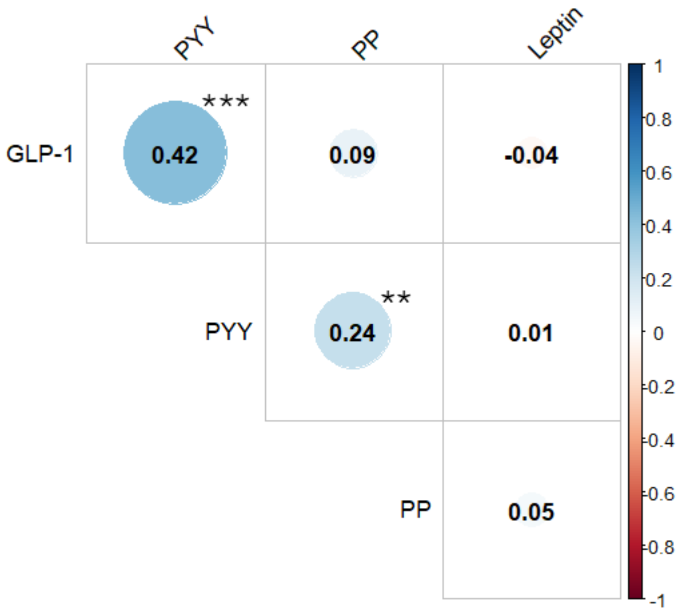

eFigure 2 - Spearman rank correlation coefficients between the cord blood appetite hormone levels. * p < 0.05;** p<0.01; *** p < 0.001. Abbreviations: GLP-1; Glucagon-like-peptide-1, PYY; Peptide tyrosine tyrosine, PP; Pancreatic polypeptide

© 2025 De Ruyter et al. *JAMA Netw Open.*

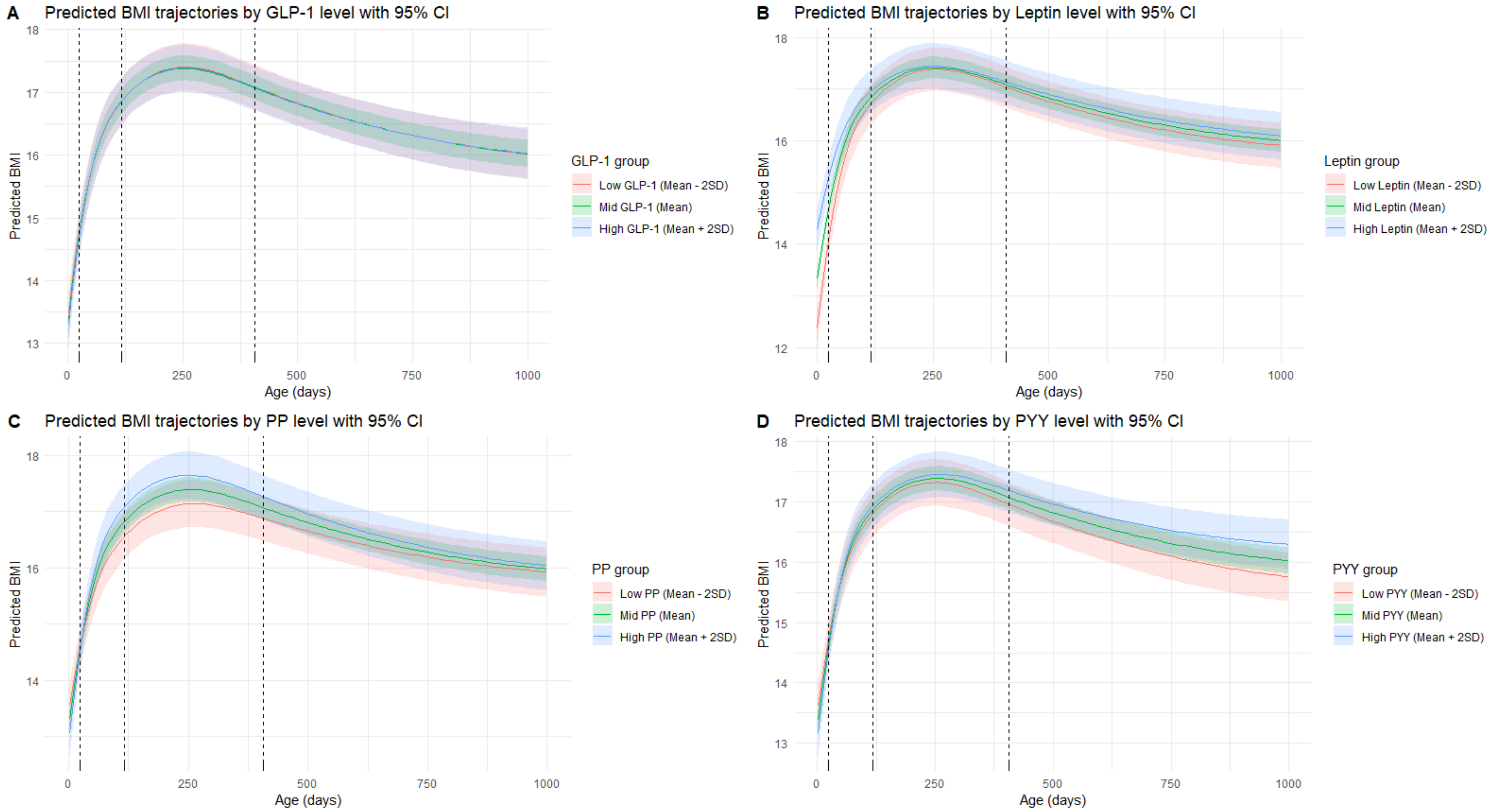

eFigure 3 - Predicted BMI trajectories by appetite hormone levels using spline-based mixed models. Abbreviations: GLP-1; Glucagon-like-peptide-1, PYY; Peptide tyrosine tyrosine, PP; Pancreatic polypeptide, SD;Standard deviation

© 2025 De Ruyter et al. *JAMA Netw Open.*

3. SUPPLEMENTARY REFERENCES

1. United Nations Educational, S. and C. Organization. International standard classification of education : ISCED 2011. https://uis.unesco.org/sites/default/files/documents/international-standard-classification-of-education-isced-2011-en.pdf (2012).

2. Adrian, T. E. *et al.* Human distribution and release of a putative new gut hormone, peptide YY. *Gastroenterology* **89**, 1070–1077 (1985).

3. Kreymann, B., Williams, G., Ghatei, M. A. & Bloom, S. R. Glucagon-like-peptide-1 7-36: A physiological incretin in man. *Lancet* **2**, 1300–1304 (1987).

4. *Package 'Anthro'*. https://github.com/worldhealthorganization/anthro (2023).

5. Handakas, E. *et al.* Cord blood metabolic signatures predictive of childhood overweight and rapid growth. *Int J Obes* **45**, 2252–2260 (2021).

© 2025 De Ruyter et al. *JAMA Netw Open.*
