# Supplementary material for: Cord Blood Appetite Hormones and Early-Life Growth and Childhood Adiposity in the ENVIRONAGE Cohort
Source: JAMA Netw Open. 2025 Nov 6;8(11):e2542140. doi: 10.1001/jamanetworkopen.2025.42140 (PMC12593129; doi:10.1001/jamanetworkopen.2025.42140)
Supplement: Supplement 2. — Data Sharing Statement [file jamanetwopen-e2542140-s002.pdf]

Data Sharing Statement

De Ruyter. Cord Blood Appetite Hormones and Early-Life Growth and Childhood Adiposity in the ENVIRONAGE Cohort. *JAMA Netw Open*. Published November 06, 2025. doi:10.1001/jamanetworkopen.2025.42140

Data
Data available: Yes
Data types: Deidentified participant data
How to access data: via tim.nawrot@uhasselt.be
When available: With publication

Supporting Documents
Document types: None

Additional Information
Who can access the data: researchers whose proposed use of the data has been approved
Types of analyses: meta-analysis
Mechanisms of data availability: after approval of a proposal
